# Population pharmacokinetic modeling of tacrolimus using real-world data for precision dosing in pediatric liver transplant recipients younger than 36 months during the immediate postoperative period

**DOI:** 10.3389/fphar.2026.1896778

**Published:** 2026-09-23

**Authors:** Soo-Min Jung, Minkyu Kim, Shi-Hyang Lee, Seungchan Choi, Kyun-Seop Bae, Hyeong-Seok Lim

**Affiliations:** 1 Department of Clinical Pharmacology and Therapeutics, Asan Medical Center, University of Ulsan College of Medicine, Seoul, Republic of Korea; 2 Department of Pediatric Surgery, Asan Medical Center Children’s Hospital, University of Ulsan College of Medicine, Seoul, Republic of Korea

**Keywords:** model-informed precision dosing, pediatric liver transplantation, population pharmacokinetics, real-world data, tacrolimus

## Abstract

**Background:**

Tacrolimus dosing during the immediate postoperative period after pediatric liver transplantation is challenging because clearance can change rapidly as graft function recovers, while conventional weight-based dosing provides limited guidance for incorporating measured concentrations into subsequent doses. We developed a population pharmacokinetic model of tacrolimus in recipients younger than 36 months and evaluated its application to model-informed precision dosing (MIPD).

**Methods:**

Routine therapeutic drug monitoring data collected through postoperative day 11 were analyzed using nonlinear mixed-effects modeling. Sampling occasions were stratified into six predefined postoperative intervals, and inter-occasion variability in apparent clearance was estimated separately for each occasion. Model performance was evaluated using goodness-of-fit diagnostics, an occasion-stratified prediction-corrected visual predictive check, and Monte Carlo simulation of individual concentration–time profiles. MIPD was implemented using two complementary approaches: a covariate-based equation for initial dosing and maximum a posteriori Bayesian forecasting for subsequent dose refinement. Forward predictive performance was also evaluated by forecasting the trough concentration at the next occasion using covariates alone or in combination with one or two preceding trough measurements.

**Results:**

A total of 731 tacrolimus concentrations from 92 patients were analyzed. A one-compartment model with first-order absorption adequately described the data. Clearance decreased with increasing total bilirubin and aspartate aminotransferase, whereas volume of distribution increased with age. Inter-occasion variability in clearance varied across the postoperative course, from a coefficient of variation of 123.8% on postoperative day (POD) 2%–10.7% on POD 5–6, before increasing to 38.8% on POD 9–11. Monte Carlo simulation placed 89.2% of observed concentrations within the 90% prediction interval. Moreover, incorporating one preceding trough reduced the mean absolute error of next-occasion predictions by approximately one-fifth, while incorporating two preceding troughs reduced it by approximately one-third, according to the performance validation.

**Conclusion:**

Tacrolimus clearance in pediatric liver transplant recipients younger than 36 months is characterized by substantial occasion-specific variability during the immediate postoperative period, consistent with dynamic changes in graft function. Dose individualization should therefore incorporate occasion-specific therapeutic drug monitoring data. The developed model provides a quantitative framework for MIPD, implemented here through a publicly accessible web application (https://smjlab.shinyapps.io/fk506_frontiers/).

## 1 Introduction

Tacrolimus is the cornerstone immunosuppressive agent for the prevention of acute rejection following solid organ transplantation (Guy-Viterbo et al., 2013). As a calcineurin inhibitor, TAC has a narrow therapeutic window and exhibits substantial intra- and inter-individual pharmacokinetic (PK) variability. Precise control of drug exposure is therefore critical: subtherapeutic concentrations increase the risk of acute rejection, whereas supratherapeutic levels are associated with serious adverse effects (Staatz and Tett, 2004; Bentata, 2020). Accordingly, therapeutic drug monitoring (TDM) is considered essential to optimize its clinical use.

In routine clinical practice, TDM for tacrolimus is primarily based on trough concentrations (C_0_). However, this approach has several important limitations. First, inaccurate timing of blood sampling is common in real-world settings and may lead to misinterpretation of drug exposure and inappropriate dose adjustments. Indeed, only a minority of samples are collected at true trough time points in clinical practice (Kirubakaran et al., 2020). Second, trough concentrations may correlate poorly with overall drug exposure, typically quantified by the area under the concentration–time curve (AUC), which is more closely associated with clinical efficacy and toxicity (Brunet et al., 2019). Third, conventional TDM lacks a clear pharmacological framework: it relies on poorly defined therapeutic ranges and does not provide explicit guidance for translating measured concentrations into individualized dose recommendations, leaving dose adjustment largely empirical (Holford et al., 2022).

To address these limitations, model-based approaches such as population PK modeling and Target Concentration Intervention (TCI) have been proposed. Population PK models characterize typical drug disposition and its variability within a population, while identifying patient-specific covariates that influence PK parameters. When combined with Bayesian forecasting, these models enable estimation of individual PK parameters using sparse and randomly timed concentration data. This approach allows for precise dose individualization without requiring strictly timed sampling or steady-state conditions, thereby facilitating the practical implementation of TCI in routine care (Kirubakaran et al., 2020; Holford et al., 2022).

The need for such approaches is particularly pronounced in pediatric liver transplant recipients, especially during the early postoperative period. In this setting, tacrolimus PK is highly unstable due to rapid physiological changes, including maturation of metabolic pathways, fluctuations in organ function, and dynamic recovery of the transplanted liver graft (Staatz et al., 2010; Guy-Viterbo et al., 2013; Brooks et al., 2021). As a result, conventional weight-based dosing strategies and empirical dose adjustments are often inadequate for achieving target exposure in young children.

Although several population PK studies of tacrolimus have been conducted in both adult and pediatric populations, relatively few have translated these models into clinically applicable tools for individualized dosing in real-world clinical settings, particularly in infants and young children during the early post-transplant period, a setting in which such models remain limited despite high PK variability.

Therefore, the aim of this study was to develop a population PK model for TAC using real-world data from pediatric liver transplant recipients younger than 36 months in the early postoperative period. Using clinically accessible covariates, we developed a population PK model intended to support future implementation of model-informed precision dosing (MIPD) based on Bayesian forecasting, thereby providing a foundation for operationalizing the TCI paradigm in this highly vulnerable population.

## 2 Materials and methods

### 2.1 Study population and dataset

#### 2.1.1 Study design

This retrospective, single-center cohort study was conducted at Asan Medical Center Children’s Hospital between 1 January 2010, and 31 May 2023. The study protocol was approved by the Institutional Review Board of Asan Medical Center (IRB No [2023–1239]).

Pediatric patients were eligible for inclusion if they met all of the following criteria: (i) underwent primary liver transplantation, including either living-donor or deceased-donor transplantation, for biliary atresia during the study period; (ii) were younger than 36 months of age at the time of transplantation; and (iii) had at least one available whole-blood TAC concentration measurement obtained between transplantation and 36 months of age. Patients were excluded if they had (i) congenital anomalies of major organs or underlying medical conditions other than biliary atresia that could substantially affect hemodynamics or tacrolimus pharmacokinetics, or (ii) no recorded tacrolimus concentration data within the observation period.

#### 2.1.2 Patient population and data measurements

A total of 92 pediatric liver transplant recipients younger than 36 months of age were included in the analysis. Because data were collected in a real-world clinical setting, blood sampling was not performed according to a predefined schedule and sampling times varied across patients. The distributions of clinical covariates evaluated during the modeling process, including total bilirubin (TBIL), aspartate aminotransferase (AST), alanine aminotransferase (ALT), serum albumin, serum creatinine, and hematocrit, were also reported.

A total of 731 whole-blood tacrolimus concentration data obtained from 92 subjects were included in the immediate postoperative PK modeling analysis. Clinical information was retrospectively collected from the electronic medical records. The collected demographic and clinical characteristics included recipient sex, age at transplantation, body weight, height, ABO blood type, primary diagnosis, and other relevant clinical conditions. Donor and graft information included donor type (living or deceased), donor sex, transplanted hepatic segment, graft size, and donor ABO blood type. Laboratory parameters included hematologic variables (hemoglobin, hematocrit, and platelet count), coagulation profiles (prothrombin time with international normalized ratio [INR] and activated partial thromboplastin time), and clinical chemistry variables (creatinine, albumin, aspartate AST, ALT, TBIL, and direct bilirubin). When a laboratory value was not measured on the same day as a tacrolius concentration sample, the most recent prior measurement was carried forward and used for the analysis (last-observation-carried-forward method).

#### 2.1.3 Tacrolimus administration and measurement

Tacrolimus was administered orally twice daily, beginning on postoperative day (POD) one at an initial dose of 0.0375 mg/kg per dose, in accordance with the institutional pediatric liver transplantation immunosuppression protocol. As trough concentrations are defined as samples obtained immediately prior to the next scheduled dose, the earliest available trough measurement corresponded to the morning of POD 2 (approximately 12 h after the initial evening dose). Subsequent trough samples were obtained as part of routine therapeutic drug monitoring during the early postoperative period. Data collected included the administered tacrolimus dose, dates and times of administration, dates and times of blood sampling for therapeutic drug monitoring, and corresponding whole-blood tacrolimus concentrations. Whole-blood tacrolimus concentrations were measured using a chemiluminescent magnetic microparticle immunoassay (ACMIA; Affinity Column Mediated Immunometric Assay) on an automated analyzer. The lower limit of quantification of the assay was 1.0 ng/mL. Whole blood samples collected up to POD 11 were gathered.

### 2.2 Establishing population PK model

#### 2.2.1 Structural model

A one-compartment model with first-order absorption and first-order elimination was selected as the structural model. The selection was supported by the predominantly trough-enriched sampling design of the dataset and by the consistency of this structure with previously published population PK models of tacrolimus in pediatric solid organ transplant recipients (Sam et al., 2000). The model was parameterized in terms of apparent clearance (CL/F), apparent volume of distribution (V/F), and the first-order absorption rate constant (KA). Two- and three-compartment models were also explored during model development; however, these models did not yield meaningful improvements in model fit and produced unstable parameter estimates given the available data. Therefore, the simpler one-compartment structure was retained as the base model for subsequent covariate analysis. Because the available concentration data were predominantly pre-dose trough samples and contained insufficient information from the absorption phase to support reliable estimation of KA, this parameter was fixed at 4.5 h^-1^ based on previously reported values for orally administered tacrolimus in pediatric populations (Wang et al., 2019). The sensitivity of the model to this assumption was qualitatively evaluated by testing alternative fixed values within the reported literature range, which resulted in negligible changes in the estimates of CL/F and V/F.

#### 2.2.2 Fixed effect and random effect

For CL/F and V/F, a typical value representing the population estimate for a reference individual was estimated as a fixed effect, denoted by TV(CL/F) and TV(V/F), respectively. Inter-individual variability (IIV) was modeled using exponential random effects on CL/F and V/F. In addition, inter-occasion variability (IOV) on CL/F was estimated for each of six pharmacokinetic occasions (OCCPKs) stratified by postoperative day: OCCPK 1 (POD 2), OCCPK 2 (POD 3), OCCPK 3 (POD 4), OCCPK 4 (POD 5–6), OCCPK 5 (POD 7–8), and OCCPK 6 (POD 9–11). Each occasion corresponded to a morning trough measurement, reflecting the routine therapeutic drug monitoring schedule during the early postoperative period. In the final model, CL/F was modeled using an exponential random-effects model, incorporating both IIV ( $\eta_{CL}$ ) and IOV ( $\eta_{OCC}$ ), as shown in Equation 1.

$$CL/F=TV\left(CL/F\right)\cdot \exp\left(\eta_{CL}+\eta_{OCC}\right)$$ (1)

V/F was modeled using an exponential random-effects model, incorporating IIV ( $\eta_{V}$ ), as shown in Equation 2.

$$V/F=TV\left(V/F\right)\cdot \exp\left(\eta_{V}\right)$$ (2)

KA was estimated as a fixed effect without associated inter-individual or inter-occasion variability. All random effects ( $\eta_{CL}$ , $\eta_{V}$ and $\eta_{OCC}$ ) were assumed to be normally distributed with a mean of zero and a variance estimated during model development.

#### 2.2.3 Residual variability

Residual unexplained variability was described using a combined additive and proportional error model. Specifically, the residual standard deviation ( $\left.W\right)$ was modeled as shown in Equation 3.

$$W=\sqrt{\sigma_{\text{add}}^{2}+\sigma_{\text{prop}}^{2}\times {\text{IPRED}}^{2}}$$ (3)

where $\sigma_{add}$ : the additive error, $\sigma_{prop}$ : the proportional error, IPRED: the individual predicted value from the model. Residual variability was assumed to follow a standard normal distribution. The additive component is expressed in concentration units (ng/mL), whereas the proportional component is expressed as a percentage.

#### 2.2.4 Covariate analysis

Each covariate was tested on CL/F and V/F as biologically appropriate using a power model with a reference value, by using the forward inclusion and backward selection method. In forward inclusion, covariates were retained if their addition reduced the objective function value (OFV) by more than 3.84 (p < 0.05, 1 degree-of-freedom), whereas in backward elimination, covariates were retained only if their removal increased OFV by more than 6.63 (p < 0.01, 1 degree-of-freedom).

For continuous covariates, the effect of covariate on a parameter $\left(\theta \right)$ was modeled as shown in Equation 4.

$$\theta_{i}=\theta_{TYP}\times {\left(\frac{{\text{COV}}_{i}}{{\text{COV}}_{r\text{ef}}}\right)}^{\theta_{\text{COV}}}$$ (4)

where $\theta_{i}$ : the parameter value for the i -th individual, $\theta_{TYP}$ : the typical parameter value, $\theta_{\text{COV}}$ : the covariate effect parameter, ${\text{COV}}_{i}$ : the covariate value for the i -th individual, ${\text{COV}}_{\text{ref}}$ : the reference covariate value of the study population.

For categorical covariates, the effect of a covariate on a parameter $\left(\theta \right)$ was modeled as shown in Equation 5.

$$\theta_{i}=\theta_{TYP}\times \left(1+\theta_{\text{COV}}\times {\text{CAT}}_{i}\right)$$ (5)

where $\theta_{i}$ : the parameter value of i -th individual, $\theta_{TYP}$ : the typical parameter value, $\theta_{\text{COV}}$ : typical parameter effect of covariate, ${\text{CAT}}_{i}$ : the categorical covariate value for the i -th individual, coded as an indicator variable.

Inter-individual and inter-occasion variabilities were quantified as variance components (ω^2^). Clinical interpretation was based on the apparent coefficient of variation (CV, %), derived under the assumption of a log-normal distribution as CV = √(exp (ω^2^) − 1) × 100. Relative standard error (RSE) was reported for each parameter as a measure of estimation precision. η-shrinkage was calculated as 1 − SD (η_EBE)/ω to assess the extent to which individual empirical Bayes estimates (EBE) were informed by the observed data rather than by the population prior.

### 2.3 Assessing population PK model

#### 2.3.1 Goodness-of-fit diagnostic plots

Goodness-of-fit plots were evaluated to assess model adequacy. Five diagnostics were inspected: observed concentrations versus population predictions (PRED) and versus individual predictions (IPRED), examined for systematic deviation from the line of identity; and conditional weighted residuals (CWRES) plotted against population predictions and against time since first dose, examined for trends in location or spread indicating structural or residual-error model misspecification.

#### 2.3.2 Prediction-corrected visual predictive check stratified by OCCPK

The predictive performance of the final model was evaluated using a prediction-corrected VPC (pcVPC) based on 1,000 simulations of the original dataset (Bergstrand et al., 2011). The pcVPC was stratified by pharmacokinetic occasion (OCCPK 1–6), and prediction correction was applied within each stratum using the median population prediction of that occasion as the reference, so that between-occasion differences in typical prediction were preserved. Because the sampling design was trough-based and each subject contributed only one or two observations per occasion, occasions were considered as the binning uni. For each simulated replicate, the 5th, 50th and 95th percentiles of the prediction-corrected concentrations were computed within each occasion, and their 2.5th and 97.5th percentiles across replicates were taken as the 95% confidence intervals of the simulated percentiles. Predictive performance was considered adequate if the corresponding observed percentiles fell within these intervals on each occasion.

#### 2.3.3 Evaluating individual predictive performance by Monte Carlo simulation

The individual-level predictive performance of the final model was evaluated by Monte Carlo simulation. For each of the 92 subjects, a time grid was constructed by inserting observation records at 1-h intervals from time zero to the subject’s last recorded time point (up to 240 h), while preserving the subject’s dosing history. Time-varying covariates were carried forward from the most recent preceding measurement to the dummy records using a last-observation-carried-forward approach.

Using this dataset, 250 replicate concentration-time profiles per subject were generated in NONMEM, with all final model parameters—including the structural parameters, covariate effects, inter-individual and inter-occasion variance components, and residual error parameters—fixed to their estimated values. At each time point, the median and the 5th and 95th percentiles of the simulated concentrations were derived across the 250 replicates to construct a subject- and time-specific prediction interval. Observed tacrolimus concentrations were then compared against the corresponding prediction interval, and the proportion of observations falling within the 5th–95th interval was calculated overall and stratified by pharmacokinetic occasion (OCCPK) as a measure of predictive coverage.

### 2.4 Demonstrating model-informed precision dosing method

The PK model was then used for developing model-informed dose individualization platform as the realized MIPD. Two approaches for dose selection were used: dose recommendation based on covariates and Bayesian forecasting.

#### 2.4.1 Dose recommendation based on covariates

The trough concentration attained at steady state is determined by clearance, volume of distribution, and the dosing interval, and the dose D (mg) required to achieve a target trough concentration $C_{trough}$ (ng/mL) over an interval τ (h) is shown in Equation 6.

$$D\;\left(mg\right)=\frac{C_{trough}\cdot \left(k_{a}-k\right)}{k_{a}\cdot \left[\frac{e^{-k\tau }}{1-e^{-k\tau }}-\frac{e^{-k_{a}\tau }}{1-e^{-k_{a}\tau }}\right]}\cdot \frac{\left(V/F\right)}{1000}$$ (6)

where CL/F (L/h) and V/F (L) are determined by the final PK model including covariates, and k is terminal rate constant defined as $\left(CL/F\right)/\left(V/F\right)$ . A target trough of 10 ng/mL was used throughout (Spada et al., 2009).

#### 2.4.2 Bayesian forecasting

Once therapeutic drug monitoring data were given, individual random effects — η on CL/F and V/F, and one inter-occasion deviation κ per sampled occasion—were estimated by maximum a posteriori (MAP) estimation, minimizing the objective function value $OFV\left(\eta ,\kappa \right)$ shown in Equation 7.

$$OFV\left(\eta ,\kappa \right)=\sum_{i}\left[\frac{{\left(C_{i,obs}-C_{i,pred}\right)}^{2}}{W_{i}^{2}}+\ln W_{i}^{2}\right]+\eta^{T}\Omega^{-1}\eta +\sum_{j}\frac{\kappa_{j}^{2}}{\omega_{j}^{2}}$$ (7)

where $C_{i,obs}$ is the observed data for i -th individual, $C_{i,pred}$ : the model predicted value for i -th individual, Ω : the inter-individual variance-covariance matrix, $\omega_{j}^{2}$ : the inter-occasion variance of j -th occasion, $W_{i}$ : the residual standard deviation for i -th individual and $W_{i}^{2}$ is expressed in Equation 8.

$$W_{i}^{2}=\sigma_{add}^{2}+{\left(\sigma_{prop}\cdot C_{i,pred}\right)}^{2}$$ (8)

#### 2.4.3 Demonstrating predictive performance of MIPD

The estimated result using this model was illustrated in four representative subjects. These four subjects were selected to reflect the observed range of graft function and discrepancies between covariate-based and observed trough concentrations. Its predictive performance was then evaluated across the cohort by forecasting the trough concentration at the next occasion under the dose administered, comparing predictions informed by covariates alone with those additionally informed by one or two preceding trough measurements. Performance was summarized as mean absolute error, root mean squared error, mean absolute percentage error, and the coverage of the 90% prediction interval. Prediction intervals used to assess coverage were obtained by Monte Carlo simulation with 2,000 replicates, drawing the individual random effects from their posterior distribution conditional on the observed concentrations, together with the inter-occasion deviation of the upcoming occasion and residual unexplained variability.

The entire procedure was implemented as a web-based application, which assigns the pharmacokinetic occasion from the postoperative day, estimates the individual random effects from the TDM data, and returns the dose required to attain the target trough at the next occasion. The web application is publicly accessible (https://smjlab.shinyapps.io/fk506_frontiers/).

### 2.5 Software

Population PK analysis was performed using NONMEM version 7.6 (Icon Development Solutions, Ellicott City, MD, United States) with the first-order conditional estimation method with interaction (FOCE-I). Data manipulation, graphical diagnostics, and post-processing of NONMEM outputs were performed using R version 4.5.2 and the web-based dose calculator was implemented with the shiny package. All simulations were performed with fixed random seeds (2026) to ensure reproducibility.

## 3 Results

### 3.1 Patient characteristics and analyzed data

The patient demographics and covariate characteristics of the study population are summarized in Table 1, with repeated laboratory measurements reported as subject-level medians. A total of 92 pediatric patients who underwent liver transplantation were included in this PK analysis. The median patient age was 11.7 months (median age at surgery, 11.65 months). Tacrolimus concentration data are reported in Table 2 in terms of number of samples, median, minimum and maximum values. Sampling occasions were stratified into six predefined postoperative occasions (OCCPK 1–6), corresponding to POD 2 (OCCPK 1), when the first trough sample is obtained, through POD 9–11 (OCCPK 6).

**TABLE 1:** Demographic and clinical characteristics of the study population, summarized as the distribution of subject-level values.

Clinical characteristics	Subject total ( N = 92)
Continuous variables	Median (min - max)
Demographics	
Age (months)	11.70 (3.30–35.50)
Age at surgery (months)	11.65 (3.20–35.40)
Body weight (kg)	8.95 (4.30–14.40)
Graft weight (g)	287.50 (120.00–915.00)
Hematologic measurements	
Hemoglobin (g/dL)	9.00 (7.05–11.70)
Hematocrit (%)	27.45 (21.40–34.10)
Hepatic function measurements	
Total bilirubin (mg/dL)	2.30 (0.80–10.60)
Direct bilirubin (mg/dL)	1.70 (0.20–9.00)
Albumin (g/dL)	3.15 (2.20–4.75)
Aspartate aminotransferase (AST) (U/L)	68.00 (26.00–461.00)
Alanine aminotransferase (ALT) (U/L)	165.00 (49.00–1095.50)
Renal Function measurements	
Creatinine (mg/dL)	0.17 (0.10–1.70)
Categorical variables	Number (%)
Sex	
Female	54 (58.7%)
Male	38 (41.3%)
Blood type	
A+	31 (33.7%)
B+	24 (26.1%)
AB+	10 (10.9%)
O+	27 (29.3%)

Continuous variables are presented as median (minimum–maximum) and categorical variables as number (percentage). Demographic variables are summarized at the subject level (N = 92) and laboratory covariates are summarized across all records included in the analysis and therefore reflect their distribution over the analysis period. For measurements of continuous variables repeated during the analysis, each subject is represented by the median of that subject’s values; the summary therefore describes the distribution of subject-level medians. Individual observations spanned wider ranges; for example, AST from 17 to 8,781 U/L and total bilirubin from 0.4 to 29.0 mg/dL.

**TABLE 2:** Distribution of tacrolimus concentration measurements.

OCCPK	POD	Number of DVs	Median (min - max)
1	2	77	4.0 (1.0–14.9)
2	3	74	7.55 (1.3–31.4)
3	4	79	9.2 (2.3–28.0)
4	5	85	9.7 (1.1–22.4)
	6	56	9.3 (3.4–24.9)
5	7	78	8.9 (1.3–28.5)
	8	69	8.3 (2.2–24.6)
6	9	75	8.3 (2.2–21)
	10	65	8.8 (3.5–20.8)
	11	73	8.2 (3.2–20)
**Total**		731	​

Pharmacokinetic observations are stratified by pharmacokinetic occasion (OCCPK), each of which comprises one or more postoperative days as shown; tacrolimus was initiated on POD, one and the earliest trough sample was obtained on the morning of POD, 2; DV, dependent variable (observed tacrolimus concentration); OCCPK, pharmacokinetic occasion; POD, postoperative day.

### 3.2 Covariate screening

Univariate screening was performed for all candidate covariates on CL/F and V/F from one-compartment model with first-order absorption and first-order elimination, with KA fixed. The results are shown in Supplementary Tables S1, S2. For CL/F, the largest reduction in the objective function value was produced by total bilirubin (ΔOFV = −86.71), followed by alanine aminotransferase (ΔOFV = −62.88), aspartate aminotransferase (ΔOFV = −35.94), serum albumin (ΔOFV = −13.31) and serum creatinine (ΔOFV = −10.72). In contrast, demographic covariates produced negligible reductions and failed to reach the prespecified inclusion threshold of ΔOFV < −3.84, including graft weight (ΔOFV = −1.66), body weight (ΔOFV = −1.30), age (ΔOFV = −0.28), and age at operation (ΔOFV = −0.04). For V/F, no covariate reached the inclusion threshold; body weight produced the largest reduction (ΔOFV = −2.56), followed by alanine aminotransferase (ΔOFV = −1.75), total bilirubin (ΔOFV = −1.29), and age (ΔOFV = −1.10).

Total bilirubin, which produced the greatest single-covariate improvement, was carried forward as the first covariate on CL/F. In the subsequent forward step, addition of serum albumin produced the largest further reduction (ΔOFV = −11.32), followed by alanine aminotransferase (ΔOFV = −10.13), aspartate aminotransferase (ΔOFV = −8.06), and serum creatinine (ΔOFV = −5.24). AST was retained in preference to ALT because the two transaminases are highly correlated markers of hepatocellular injury, and their concurrent inclusion would introduce multicollinearity problem.

### 3.3 Final model selection

The results of forward inclusion and backward elimination are summarized in Table 3. Inclusion of age on V/F resulted in a minimal reduction in OFV (ΔOFV = −2.21); however, it was retained based on biological plausibility and to support generalizability across the pediatric age range. Body weight was evaluated as a covariate on V/F but resulted in a negative and biologically implausible exponent and was therefore excluded. To improve numerical stability while maintaining interpretability, the age exponent on V/F was fixed at 0.16, as estimated during forward selection rounded to two decimal places (Supplementary Table S2). The final model achieved the lowest OFV among all candidate models (OFV 2598.11; AIC 2628.78).

**TABLE 3:** Covariate model selection by forward inclusion and backward elimination.

Step	Model description	OFV	ΔOFV	AIC	ΔAIC
Forward inclusion					
Step 0	Base model (no covariate)	2,695.32	​	2,721.82	​
Step 1	CL/F ∼ TBIL	2,608.61	−86.71	2,637.20	−84.62
Step 2	CL/F ∼ TBIL + AST	2,600.56	−8.06	2,631.23	−5.97
Step 3	CL/F ∼ TBIL + AST and V/F ∼ AGE	2,598.35	−2.21	2,631.11	−0.12
Full model	CL/F ∼ TBIL + AST and V/F ∼ AGE (fixed covariate effect value)	2,598.11	​	2,628.78	​
Backward elimination					
Step 1	Final model − TBIL (CL/F ∼ AST only)	2,653.36	55.25	2,681.94	53.16
Step 2	Final model − AST (CL/F ∼ TBIL only)	2,605.64	7.53	2,634.23	5.45

After covariate screening, the final model including the covariates was verified with forward inclusion and backward elimination criteria. Model selection was based on ΔOFV, and AIC is reported as a supplementary metric. For Full Model, the covariate effect ( θ ) is fixed at the value estimated from Step 3. OFV, objective function value; ΔOFV, difference of OFV compared to the base model’s OFV in forward inclusion and to the full model’s OFV in backward elimination; AIC, akaike information criterion; ΔAIC, difference of AIC compared to the base model’s AIC in forward inclusion and to the full model’s AIC in backward elimination. AIC, akaike information criterion; AST, aspartate aminotransferase; CL/F, apparent clearance; OFV, objective function value; TBIL, total bilirubin; V/F, apparent volume of distribution.

Backward elimination from the full model confirmed retention of the biochemical covariates: removal of TBIL significantly worsened model fit (ΔOFV = +55.25; p < 0.001), and removal of AST also resulted in a significant deterioration (ΔOFV = +7.53; p < 0.01), exceeding the prespecified retention threshold (ΔOFV > 6.63). Successful minimization and stepwise covariate evaluation were achieved for all candidate models except the intermediate model in which both age on V/F and the age exponent were simultaneously estimated, supporting the decision to fix this parameter value in the final model.

### 3.4 Final model parameter estimates

#### 3.4.1 Fixed-effect parameters

The final population PK parameter estimates, together with their RSE and 95% confidence intervals, are presented in Table 4. The typical values of CL/F, or TV(CL/F), and V/F, or TV(V/F), were 4.85 L/h (RSE 6.0%; 95% CI, 4.27–5.44) and 124 L (RSE 9.9%; 95% CI, 99.4–148.5), respectively, both estimated with high precision. The absorption rate constant was fixed at 4.5 h^-1^.

**TABLE 4:** Fixed-effect parameter estimates and residual error of the final PK model.

Parameter (units)	Description	Estimate [%RSE]	95% CI
TV(CL/F) (L/h)	Typical apparent clearance	4.85 [6.0]	4.27–5.44
TV(V/F) (L)	Typical apparent volume of distribution	123.9 [9.9]	99.4–148.5
KA (h^-1^)	Absorption rate constant	4.5 (fixed)	-
$\theta_{TBIL}$ on CL/F	Covariate effect ( θ ) in the negative power model, as $CL/F\times {\left(TBIL/2\right)}^{-\theta }$	0.353 [14.0]	0.25–0.45
$\theta_{AST}$ on CL/F	Covariate effect ( θ ) in the negative power model, as $CL/F\times {\left(AST/60\right)}^{-\theta }$	0.106 [46.7]	0.007–0.205
$\theta_{AGE}$ on V/F	Covariate effect ( θ ) in the fixed power model, as $V/F\times {\left(AGE/12\right)}^{\theta }$	0.16 (fixed)	-
Residual error			
$\sigma_{add}$ (ng/mL)	Additive error	1.37 [19.0]	0.85–1.89
$\sigma_{prop}$ (%)	Proportional error	0.206 [7.8]	0.17–0.24

Estimates are presented with their relative standard error (RSE, %) and 95% confidence intervals calculated as the point estimate ± 1.96 × standard error. Typical apparent clearance and volume of distribution are estimated values at reference covariate values. The age effect on V/F was fixed at the value obtained during forward covariate selection. Residual unexplained variability was described by a combined additive and proportional error model. AST, aspartate aminotransferase; CI, confidence interval; KA, absorption rate constant; RSE, relative standard error; TBIL, total bilirubin.

TBIL and AST were identified as covariates on TV(CL/F), with estimated power exponents of 0.353 (RSE 14.0%; 95% CI, 0.25–0.45) and 0.106 (RSE 46.7%; 95% CI, 0.007–0.205), respectively. Both covariate effects were incorporated into the structural model using negative exponents, indicating that elevated TBIL and AST were associated with reduced TV(CL/F), consistent with impaired graft function. The covariate effect of age on TV(V/F) was modeled as a power function, with the exponent fixed at 0.16, reflecting a modest positive relationship between age and TV(V/F) in the pediatric population. The final structural models for TV(CL/F) and TV(V/F) are shown in Equations 9 and 10, respectively.

$$\text{TV}\left(\text{CL}/F\right)\;\left(L/h\right)=4.85\times {\left(\frac{\text{TBIL}}{2}\right)}^{-0.353}\times {\left(\frac{\text{AST}}{60}\right)}^{-0.106}$$ (9)

$$\text{TV}\left(V/F\right)\;\left(L\right)=124\times {\left(\frac{\text{AGE}}{12}\right)}^{0.16}$$ (10)

Residual unexplained variability was characterized by a combined error model, with an additive component ( $\sigma_{add}$ ) of 1.37 ng/mL (RSE 19.0%; 95% CI, 0.85–1.89) and a proportional component ( $\sigma_{prop}$ ) of 20.6% (RSE 7.8%; 95% CI, 17.4%–23.8%).

#### 3.4.2 Inter-individual and inter-occasion variability

The estimated random-effect parameters are summarized in Table 5. Inter-individual variability in CL/F and V/F, expressed as apparent coefficient of variation, was 61.3% and 54.2% respectively, with a moderate-to-strong positive correlation between the two parameters ( r = 0.70), consistent with shared underlying influences such as body size acting on both clearance and volume of distribution.

**TABLE 5:** Inter-individual and inter-occasion variability estimates of the final PK model.

Parameter		Variance (ω^2^)	CV (%)	RSE (%)	Shrinkage
Inter-individual variability (IIV)					
CL/F		0.319	61.3	17.3	0.97
V/F		0.258	54.2	46	0.81
Correlation (CL/F–V/F)		r = 0.7			
Inter-occasion variability (IOV) on CL/F					
POD 2	OCCPK 1	0.929	123.8	24.8	0.62
POD 3	OCCPK 2	0.114	34.8	60.1	0.44
POD 4	OCCPK 3	0.034	18.7	114.8	0.36
POD 5–6	OCCPK 4	0.011	10.7	184.2	0.31
POD 7–8	OCCPK 5	0.044	21.2	78.5	0.57
POD 9–11	OCCPK 6	0.14	38.8	22.4	0.85

CV, was calculated as √(exp (ω^2^) − 1) × 100. Inter-individual variability on CL/F and V/F was estimated with a full block covariance structure by NONMEM., Inter-occasion variability was applied to CL/F across six predefined occasions (OCCPK, 1–6). η-shrinkage is expressed as a proportion (0–1). CL/F, apparent clearance; CV, coefficient of variation; OCCPK, pharmacokinetic occasion; POD, postoperative day; RSE, relative standard error; V/F, apparent volume of distribution.

Inter-occasion variability in CL/F was pronounced in the immediate postoperative period and varied across occasions. Variability was greatest at OCCPK 1 (CV 123.8%), corresponding to the first trough measurement after tacrolimus initiation, decreased progressively over subsequent postoperative days to a minimum at OCCPK 4 (CV 10.7%), and increased again by OCCPK 6 (CV 38.8%). Precision of the inter-occasion estimates was lowest at the occasions with the smallest variance (RSE 78.5%–184.2% for OCCPK 3–5), as expected when a variance component approaches zero.

η-Shrinkage was substantial for the inter-individual terms (0.97 for CL/F and 0.81 for V/F) and considerably lower for the inter-occasion terms (0.31–0.85). This pattern is consistent with the trough-enriched sampling design, in which each subject contributed only one or two observations per occasion, and is considered further in Discussion.

### 3.5 Population PK model evaluation

#### 3.5.1 Result of goodness-of-fit diagnostic plots

The standard goodness-of-fit diagnostic plots for the final model are presented in Figure 1. Individual predicted concentrations closely followed observed values along the identity line (Figure 1B), whereas population predictions exhibited considerably greater scatter (Figure 1A), reflecting the substantial inter-individual and inter-occasion variability characteristic of this clinical setting. Conditional weighted residuals were broadly distributed around zero across the range of population predictions (Figure 1C) and individual predictions (Figure 1D) and were uniformly distributed across the entire observation period (Figure 1E). Observed concentrations showed the expected dispersion around population predictions, with a narrower range of PRED reflecting the substantial shrinkage of the individual random effects, whereas the plot against individual predictions followed the line of identity closely.

**FIGURE 1:**
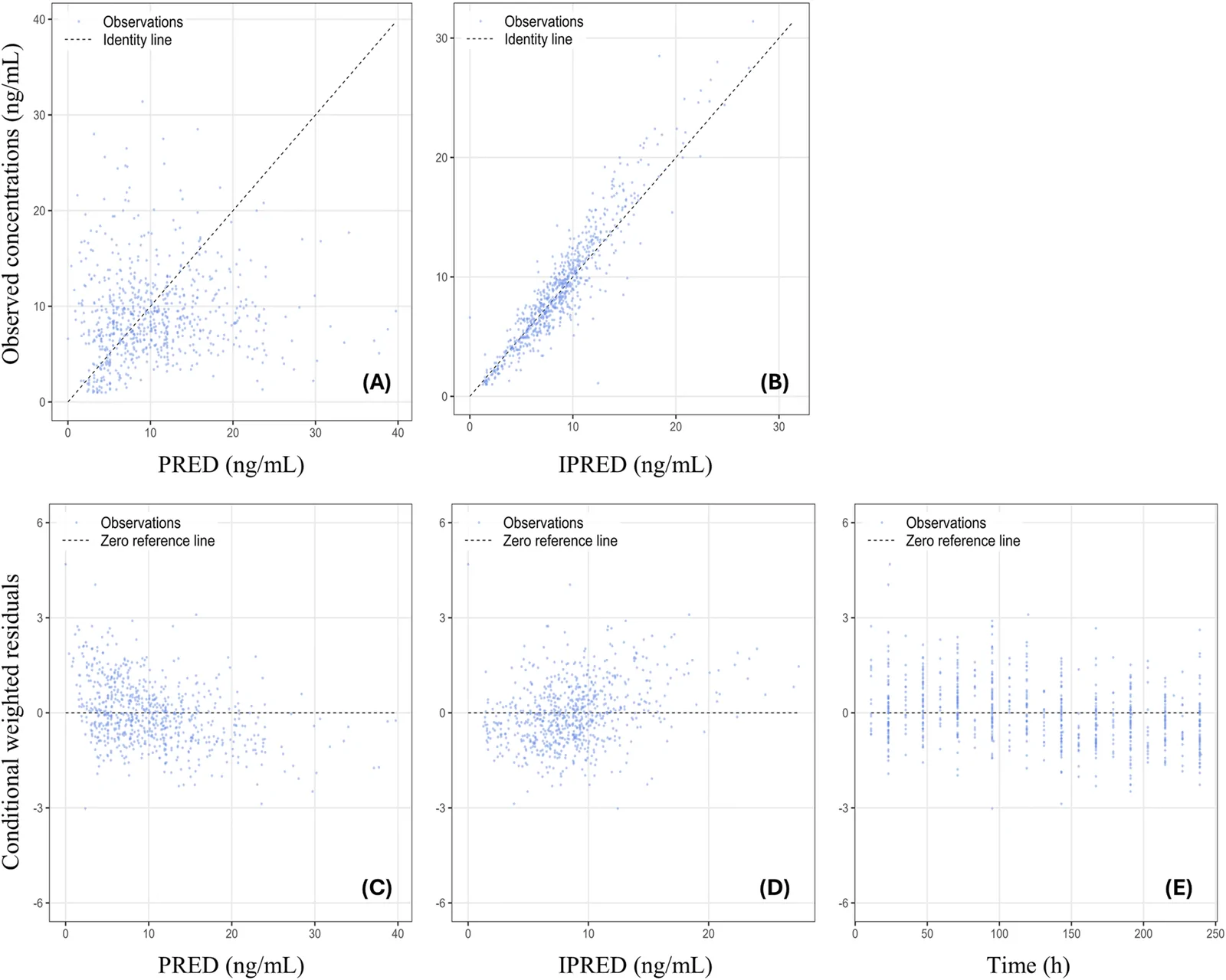
Goodness-of-fit diagnostic plots of the population PK model Observed tacrolimus concentrations were plotted against population predictions (PRED **(A)**) and individual predictions (IPRED **(B)**), with the dashed line representing the line of identity. Conditional weighted residuals (CWRES) were plotted against PRED **(C)**, IPRED **(D)**, and time after transplantation **(E)**; dashed lines represent the zero reference line. Each point represents an observed tacrolimus concentration.

#### 3.5.2 Result of occasion-stratified pcVPC

The occasion-stratified pcVPC, based on 1,000 simulated replicates of the original dataset, is shown in Figure 2. The observed median, 5^th^ percentile, and 95th percentile of TAC concentrations were contained within the corresponding 95% confidence intervals of the simulated percentiles across the entire OCCPKs (OCCPK 1–6). These results indicate that the final model adequately reproduced both the central tendency and variability of TAC concentrations in this pediatric liver transplant population, supporting its predictive performance and suitability for Bayesian forecasting and model-informed precision dosing. The proportion of simulated samples excluded due to negative prediction-corrected dependent variable (pcDV < 0) during pcVPC processing was also calculated as an indicator of simulation stability, with lower values reflecting greater model robustness This proportion was 1.95%, indicating high stability of model simulations and supporting the robustness of the final model (Bergstrand et al., 2011). The stratified check also made visible the progressive narrowing of the prediction interval across occasions, most markedly between the first and second occasions, consistent with the estimated decline in inter-occasion variability in clearance from POD 2 onward.

**FIGURE 2:**
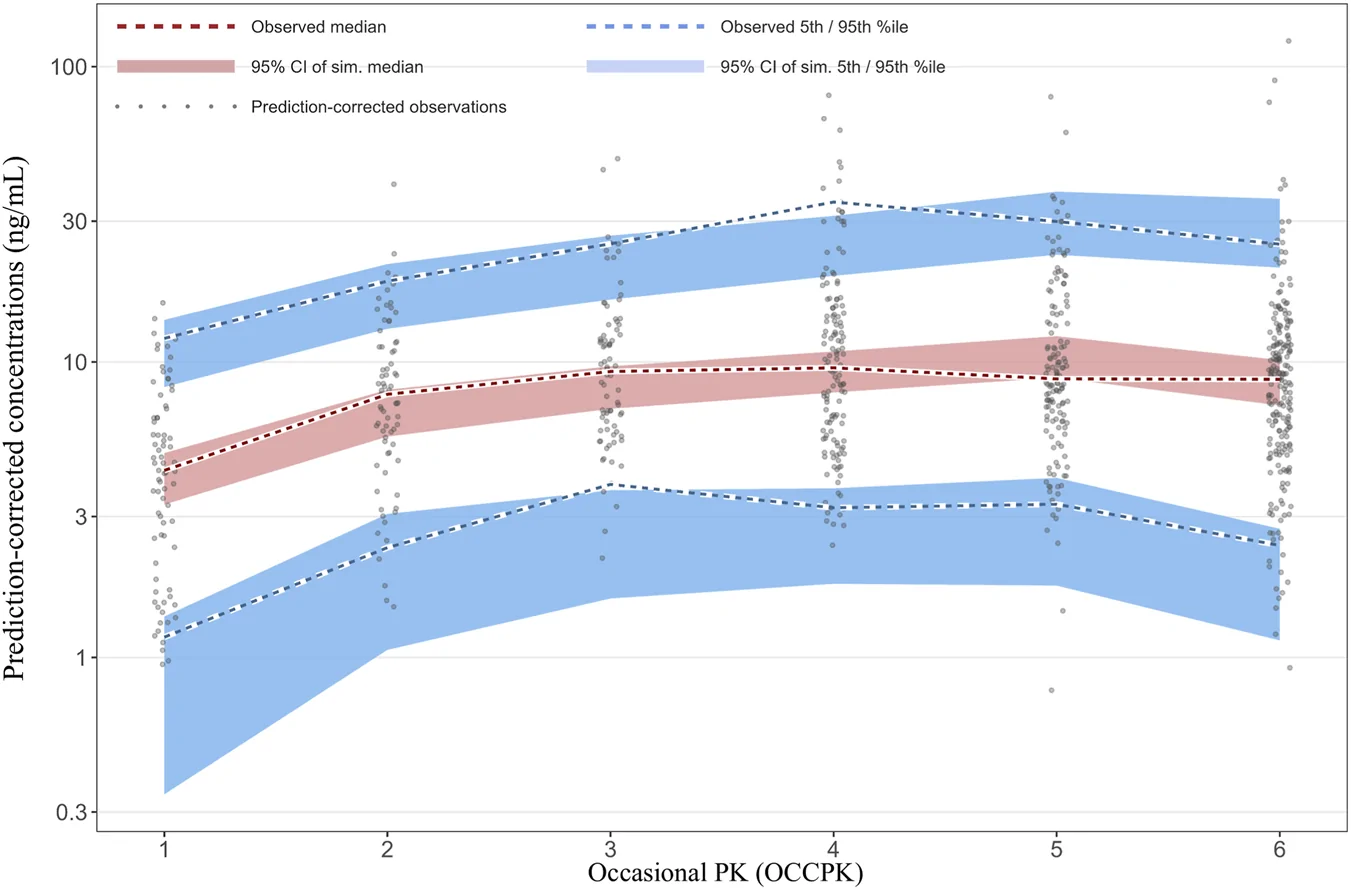
Occasion-stratified pcVPC of the population PK model. rediction-corrected observed tacrolimus concentrations are shown by gray points across the six pharmacokinetic occasions (OCCPK). The red dashed line and shaded area represent the observed median and its 95% confidence interval, respectively. The blue dashed lines and shaded areas represent the observed 5th and 95th percentiles and their corresponding 95% confidence intervals, respectively. The y-axis is presented on a logarithmic scale.

#### 3.5.3 Individual predictive performance of PK model through Monte Carlo simulation

Individual concentration-time profiles of analyzed subjects are shown in Supplementary Figure S1, including observed concentrations (DV, dependent variable), population predicted values (PRED), and individual predicted values (IPRE). Individual predictive performance was assessed by Monte Carlo simulation, in which 250 concentration-time profiles were simulated for each subject using the observed dosing history and time-varying covariates. Representative simulation results for nine subjects are shown in Figure 3, while the results for all subjects are presented in Supplementary Figure S2.

**FIGURE 3:**
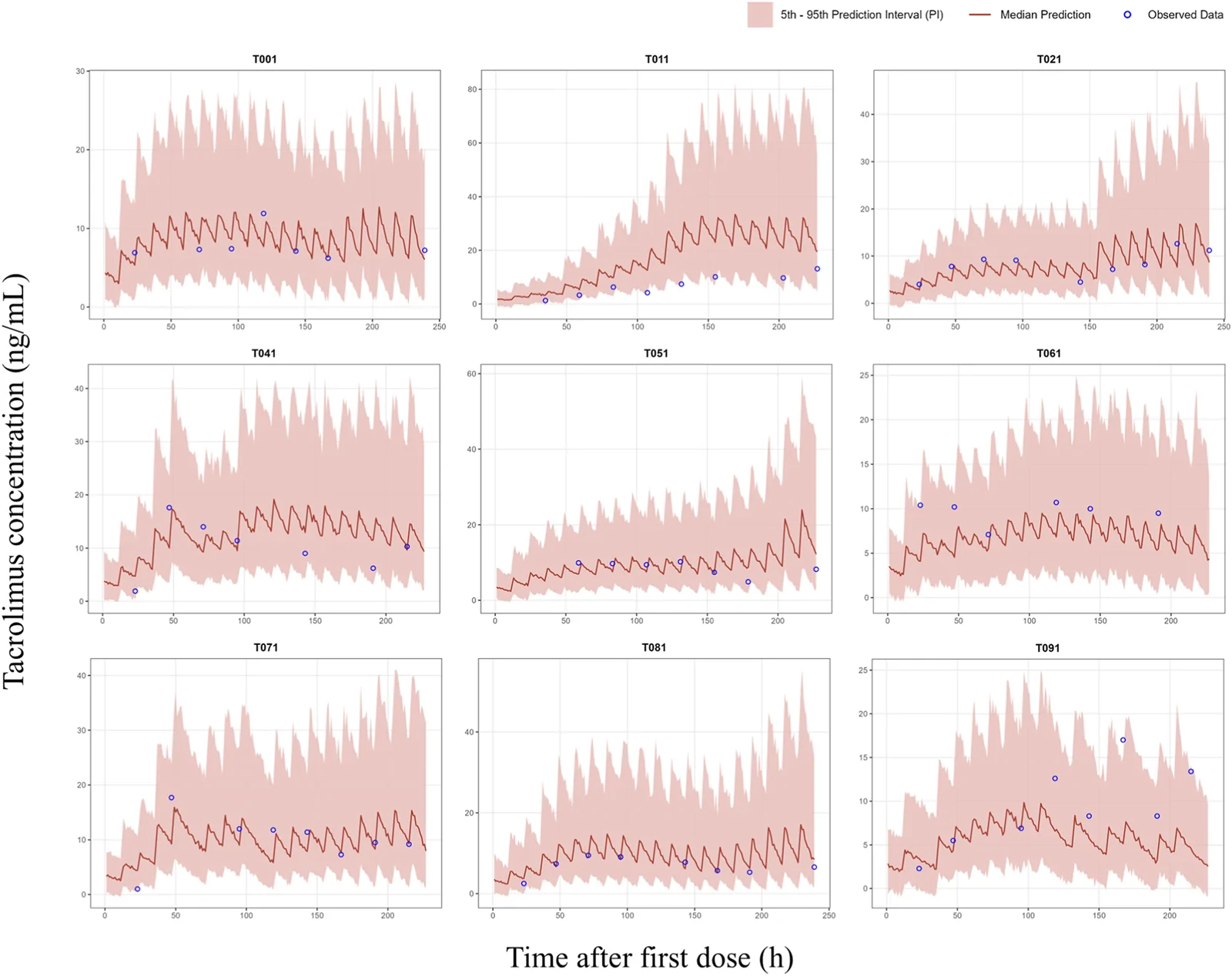
Individual predictions from Monte Carlo simulation of the population PK model. The simulated tacrolimus concentration–time profiles based on 250 Monte Carlo simulations are shown for nine representative individuals (T001, T011, T021, T041, T051, T061, T071, T081, and T091). The shaded area represents the 5th–95th percentile prediction interval of the simulated concentrations, the red line represents the median prediction, and the open blue circles represent the observed tacrolimus concentrations. The x -axis represents time after the first dose, and the y -axis represents tacrolimus concentration. Profiles for all 92 subjects are provided in Supplementary Figure S2.

The predictive coverage of the Monte Carlo simulation is summarized in Table 6. Across the 92 subjects, 652 of 731 observed concentrations (89.2%) fell within the 5th–95th prediction interval, closely approximating the 90% expected under a correctly specified model. Predictive coverage was consistent across the six PK occasions, ranging from 86.5% (OCCPK 2, POD 3) to 91.5% (OCCPK 6, POD 9–11). The simulated prediction intervals were widest during the earliest occasion, reflecting the large estimated inter-occasion variability in clearance at POD 2 (CV, 123.8%), and became progressively narrower as graft function stabilized. Coverage remained close to the nominal 90% level at both the most variable occasion (89.6% at POD 2) and the later, more stable occasion (87.2% at POD 5–6).

**TABLE 6:** Predictive coverage of the final PK model through Monte Carlo simulation.

OCCPK	POD	Observations	Within 5th - 95th PI	Coverage (%)
1	2	77	69	89.6
2	3	74	64	86.5
3	4	79	70	88.6
4	5–6	141	123	87.2
5	7–8	147	131	89.1
6	9–11	213	195	91.5
**Total**		731	652	89.2

For each of the 92 subjects, 250 concentration-time profiles were simulated using the observed dosing history and time-varying covariates, with all final model parameters fixed to their estimated values. The 5th and 95th percentiles of the simulated concentrations at each time point. Coverage was calculated as the proportion of observed concentrations falling within this interval, overall and stratified by pharmacokinetic occasion (OCCPK). Coverage close to the nominal 90% indicates adequate predictive performance of the model at the individual level. PI, prediction interval; OCCPK, pharmacokinetic occasion; POD, postoperative day.

### 3.6 Predictive performance of MIPD

#### 3.6.1 Dose estimation based on covariates

The estimated result of typical value of apparent clearance, or TV(CL/F), based on demonstrated MIPD is shown in Table 7. It incorporated the covariate values of TBIL and AST used for TV(CL/F), the corresponding model-predicted values of TV(CL/F), and the recommended dose for a target trough concentration of 10 ng/mL by OCCPK. Median TBIL fell from 4.60 mg/dL at POD 2 to 1.20 mg/dL at POD 9–11 and median AST from 188 to 45 U/L, with predicted TV(CL/F) rising at every successive occasion from 3.21 to 5.99 L/h. Because the shortening half-life also widens the peak-to-trough excursion, the dose required to maintain a trough of 10 ng/mL rose disproportionately, from 0.45 to 0.96 mg every 12 h—a 2.1-fold increase over 11 days, illustrating the clinical value of Bayesian forecasting for individualized dose adjustment.

**TABLE 7:** Median covariate values, predicted typical apparent clearance, and the covariate-based dose required for a target trough of 10 ng/mL, by pharmacokinetic occasion.

OCCPK	POD	No.	TBIL (mg/dL)	AST (U/L)	Predicted TV (CL/F) (L/h)	DV (ng/mL)	Dose for target $C_{trough}$ 10 ng/mL
1	2	77	4.60 (3.3–7.1)	188 (96–460)	3.21	4.0 (1.9–7.3)	0.45
2	3	74	4.35 (2.6–6.2)	112 (70–200)	3.45	7.5 (4.5–11.2)	0.49
3	4	79	3.50 (2.2–4.8)	77 (56–118)	3.88	9.2 (6.8–11.6)	0.56
4	5–6	141	2.80 (1.9–4.4)	72 (51–102)	4.23	9.6 (7.0–12.2)	0.62
5	7–8	147	1.80 (1.3–2.5)	54 (36–82)	5.09	8.4 (6.8–10.1)	0.78
6	9–11	213	1.20 (0.9–1.8)	45 (33–71)	5.99	8.4 (6.4–11.2)	0.96

Continuous covariates are presented as median (interquartile range). Predicted typical apparent clearance was calculated from the final covariate model as TV(CL/F) = 4.85 × (TBIL/2)^(−0.353) × (AST/60)^(−0.106), evaluated at the median covariate values of each occasion. The dose shown is that required to achieve a target trough concentration of 10 ng/mL at steady state over a 12-h dosing interval. Observed trough concentrations are shown for reference; those at the earliest occasions lie below the target range because steady state had not yet been attained. TBIL, total bilirubin; AST, aspartate aminotransferase; CL/F, apparent clearance; OCCPK, pharmacokinetic occasion; POD, postoperative day; No., number of samples; DV, dependent variable.

#### 3.6.2 Bayesian forecasting and its predictive performance of four representative subjects

To demonstrate the application of MIPD based on Bayesian forecasting, four representative subjects were selected; ID numbers T027, T054, T067, T082. Table 8 summarizes predicted parameter values and clinical data of four exemplified subjects. Bayesian forecasting substantially revised individual clearance estimates whenever observed concentrations deviated from covariate-based predictions. Three of the four representative subjects (T027, T054 and T082) required clinically meaningful dose adjustment (−60% to +29%), whereas one subject (T067) showed virtually no change.

**TABLE 8:** Bayesian forecasting of individualized tacrolimus doses in four representative subjects.

Parameters	Exemplified subjects (ID)			
	T082	T067	T054	T027
Demographics and covariates at POD 2				
Age (months)	16.5	24.6	9.4	16.2
Body weight (kg)	9.50	13.70	7.04	7.44
Total bilirubin (mg/dL)	1.9	1.7	4.5	8.2
AST (U/L)	94	93	188	6,656
Covariate-based (pre-TDM) prediction				
Typical CL/F (L/h)	4.71	4.91	3.23	1.79
Typical V/F (L)	130.4	139.0	119.2	130.0
Dose for target trough 10 ng/mL, typical parameters (mg q12 h)	0.70	0.75	0.45	0.25
Predicted POD 2 trough under the administered dose (ng/mL)	3.4	4.1	3.1	2.7
Observed therapeutic drug monitoring				
Administered dose (mg q12 h)	0.40	0.50	0.50	0.40
Observed POD 2 trough (ng/mL)	1.0	4.2	6.5	7.5
MAP Bayesian estimates				
η on CL/F	+0.212	+0.004	−0.413	−0.641
η on V/F	+0.128	+0.003	−0.449	−0.749
$\kappa_{1}$ (POD 2)	+0.639	+0.007	−0.359	−0.330
Individual CL/F at POD 2 (L/h)	11.04	4.96	1.49	0.68
Individual V/F (L)	148.2	139.5	76.1	61.5
Deviation from typical CL/F (%)	+134	+1	−54	−62
Individualized dose for the next occasion ( κ reset to 0)				
Individual CL/F (L/h)	5.82	4.93	2.14	0.94
Recommended dose (mg q12 h)	0.90	0.75	0.30	0.10
Change vs. covariate-based dose (%)	+29	0	−33	−60
Predicted trough (ng/mL)	10.2	10.3	9.9	8.1

Typical parameters were obtained from the final covariate model: TV(CL/F) = 4.85 × (TBIL/2)^(−0.353) × (AST/60)^(−0.106) and TV(V/F) = 124 × (age/12)^(0.16). Individual random effects were estimated by MAP Bayesian estimation using the single POD 2 trough concentration. Because the inter-occasion deviation is calculated independently on each occasion, κ was reset to zero when projecting the dose for the subsequent occasion. Doses were rounded to the nearest 0.05 mg. MAP, maximum a posteriori; POD, postoperative day; CL/F, apparent clearance; V/F, apparent volume of distribution.

In subject T067 (TBIL 1.7 mg/dL, AST 93 U/L) the covariate model predicted a POD 2 trough of 4.1 ng/mL under the administered dose of 0.50 mg and the observed value was 4.2 ng/mL; MAP estimation left clearance essentially unchanged (+1% versus typical) and the recommended dose was identical to that obtained from covariates alone. In the remaining three subjects the observed concentration diverged substantially from the covariate-based prediction. Subject T082, who had preserved biochemical graft function (TBIL 1.9 mg/dL, AST 94 U/L), had an observed POD 2 trough of 1.0 ng/mL against a predicted 3.4 ng/mL; MAP estimation identified a clearance 134% above the typical value, and the dose required for a target trough of 10 ng/mL rose from 0.70 to 0.90 mg every 12 h. Conversely, in subject T027, who had extreme transaminase elevation (AST 6656 U/L), the observed trough of 7.5 ng/mL exceeded the predicted 2.7 ng/mL; the MAP clearance was 62% below typical and the recommended dose fell from 0.25 to 0.10 mg every 12 h. Subject T054, close to the cohort median covariate values, showed an intermediate downward correction (clearance 54% below typical; dose 0.45 → 0.30 mg). Figure 4 illustrates individual MAP-based Bayesian estimation substantially updated population-based tacrolimus predictions according to individual observations, yielding individualized CL/F estimates.

**FIGURE 4:**
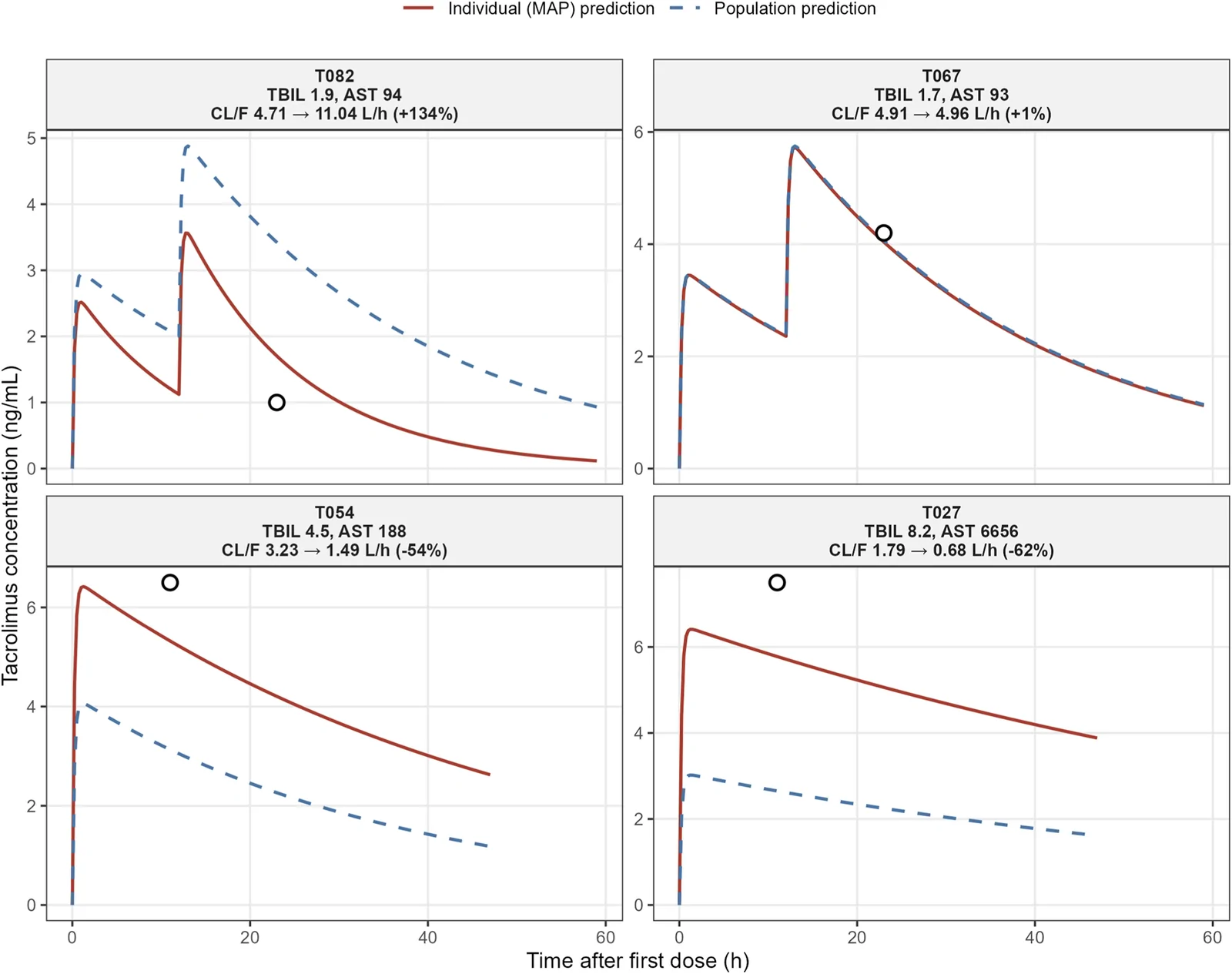
Bayesian forecasting of tacrolimus concentrations using individual PK data. Observed tacrolimus concentrations (open circles) and model-predicted concentration–time profiles are shown for four representative individuals (T027, T054, T067, and T082). The red solid line represents the individual maximum a posteriori (MAP) prediction, and the blue dashed line represents the population prediction. The corresponding changes in predicted apparent clearance (CL/F) after Bayesian forecasting are indicated for each individual, together with the values of TBIL and AST, illustrating individualized dose adjustment. The x -axis represents time after the first dose, and the y -axis represents tacrolimus concentration.

Table 9 shows the predictive performance of the models incorporating methods explained for estimating the dose for POD 3 and 4. Forecasting the POD 3 trough under the dose actually administered, the addition of a single POD 2 trough measurement to the covariate model reduced the mean absolute error from 4.0 to 3.2 ng/mL and the root mean squared error from 5.9 to 4.9 ng/mL in the 62 subjects with observations on both occasions. For the POD 4 trough, in the 54 subjects with observations at three consecutive occasions, mean absolute error fell from 4.7 ng/mL with covariates alone to 4.2 ng/mL with one preceding measurement and 3.1 ng/mL with two measurements, with mean absolute percentage error falling from 43.0% to 29.9%. The procedure is reflected in a web-based calculator (Figure 5), which reproduces the calculations described above for an individual patient at the point of care.

**TABLE 9:** Predictive performance of Bayesian forecasting.

Target occasion	Information used	No.	MAE (ng/mL)	RMSE (ng/mL)	MAPE (%)	90% PI coverage (%)
POD 3 (OCCPK 2)	Covariates only (no TDM)	62	4.0	5.9	54.4	90.3
	+ POD 2 trough	62	3.2	4.9	44.6	90.3
POD 4 (OCCPK 3)	Covariates only (no TDM)	54	4.7	6.6	43.0	88.9
	+ POD 2 trough	54	4.2	5.9	42.5	87.0
	+ POD 2 and POD 3 troughs	54	3.1	4.5	29.9	92.6

Analyses were restricted to subjects with observations at POD3 and 4. Predictions were made for the trough concentration under the dose administered, using covariates measured at the target occasion. Prediction intervals incorporate posterior uncertainty in the individual random effects, the inter-occasion variance of the target occasion, and residual unexplained variability. The number of subjects is smaller than the number of observations at the corresponding occasion in Table 6, because forward prediction requires observations at consecutive occasions within the same subject. MAE, mean absolute error; RMSE, root mean squared error; MAPE, mean absolute percentage error; PI, prediction interval; TDM, therapeutic drug monitoring; POD, postoperative day. No.; number of samples.

**FIGURE 5:**
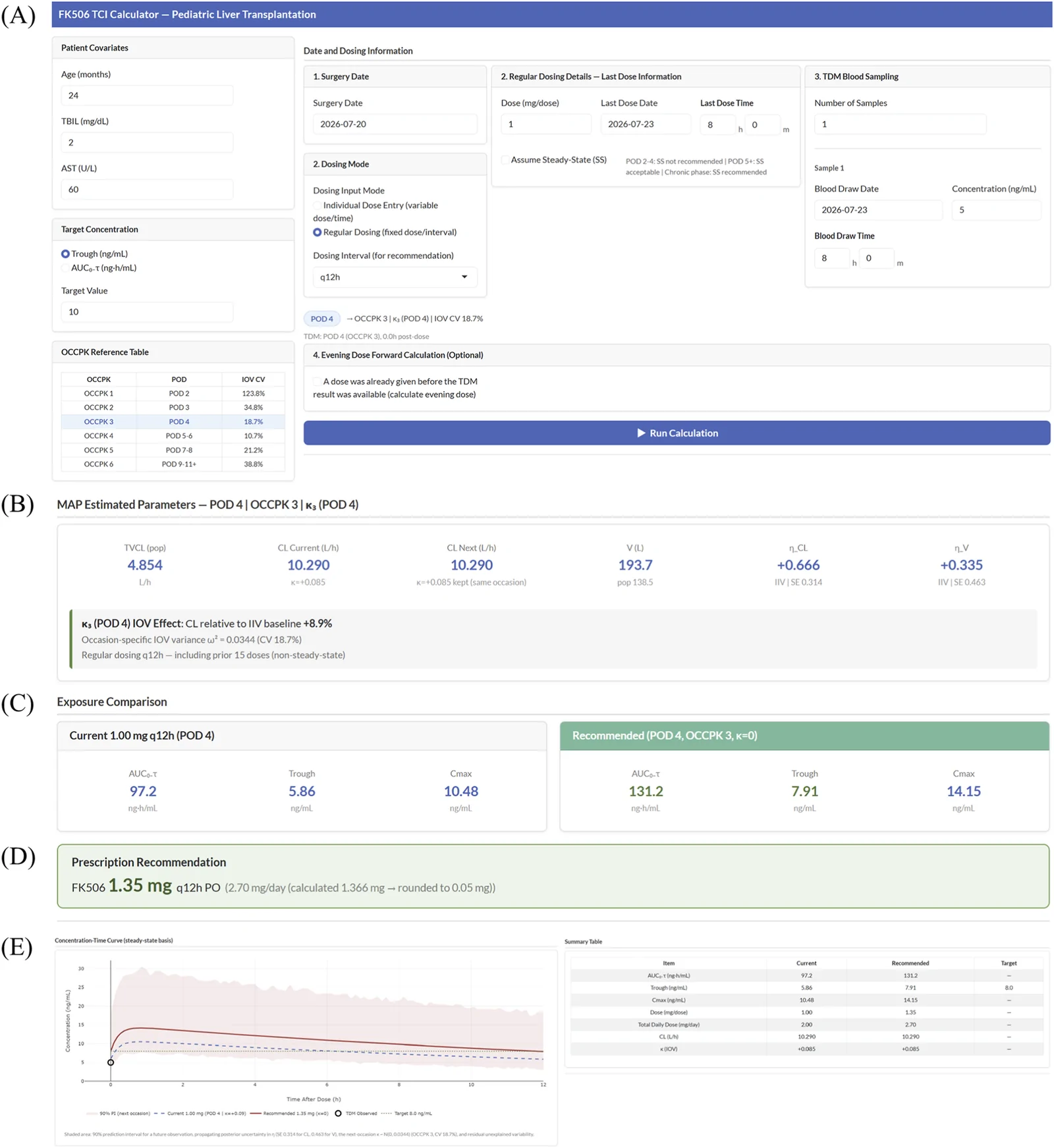
Web-based dose calculator implementing the final population pharmacokinetic model based on Bayesian forecasting **(A)** Input panel for patient covariates, dosing history, and therapeutic drug monitoring results, with the pharmacokinetic occasion assigned from the surgery date **(B)** Individual parameters estimated by maximum a posteriori (MAP) Bayesian estimation, showing the typical value, the individual estimate for the current and the upcoming occasion, and the corresponding random effects **(C)** Comparison of predicted exposure under the current and the recommended regimen **(D)** Recommended dose, rounded to the nearest 0.05 mg **(E)** Predicted concentration–time profile with the 90% prediction interval for a future observation, propagating the posterior uncertainty of the individual random effects, the inter-occasion deviation of the upcoming occasion, and residual unexplained variability.

## 4 Discussion

This study developed and evaluated a population PK model of tacrolimus in pediatric liver transplant recipients younger than 36 months of age during the immediate postoperative period in which tacrolimus dose titration is highly challenging (Barriga-Rodríguez et al., 2025). A one-compartment model with first-order absorption adequately described the observed concentration–time data, with apparent clearance influenced by TBIL and AST as dynamic markers of graft function and apparent volume of distribution scaled by age. A distinguishing feature of the model was the inclusion of IOV across six postoperative occasions (OCCPK 1–6), capturing marked PK instability during the early postoperative period. Occasions were defined from POD 2 onward, as tacrolimus was initiated on POD one and routine TDM commenced on POD 2 (Balzan et al., 2005; Hyder et al., 2013). The population PK model was evaluated with goodness-of-fit diagnostic plots, occasion-stratified pcVPC and individual prediction through Monte Carlo simulations. The predictive performance of MIPD was demonstrated by dose estimation containing covariates and exemplified with real-world clinical data and model-based prediction of four representative subjects.

The predictive validity of population PK model was supported by two independent evaluations. First, the pcVPC demonstrated that the observed median and the 5th and 95th percentiles of tacrolimus concentrations were contained within the 95% confidence intervals of the corresponding simulated percentiles across the entire observation period. Second, Monte Carlo simulations of individual concentration-time profiles, based on each subject’s dosing history and time-varying covariates, showed that 652 of 731 observed concentrations (89.2%) fell within the 5th–95th prediction interval, closely matching the 90% expected under a correctly specified model.

A notable feature of this model is the use of dynamic biochemical markers (TBIL and AST) rather than static anatomical descriptors such as graft-to-recipient weight ratio, which has been evaluated in previous pediatric studies (Lo et al., 1996). Although graft-to-recipient weight ratio provides a pre-transplant estimate of functional liver mass, it does not reflect postoperative changes in graft function (Olthoff et al., 2015). In contrast, TBIL and AST are routinely measured and better capture real-time recovery of metabolic capacity of liver (Musuamba et al., 2014).

TBIL and AST were significant covariates on CL/F, both with negative power exponents, indicating reduced clearance with increasing covariate values (Bruce et al., 1995; Lee et al., 2006). A doubling of TBIL from the reference value of 2 mg/dL was associated with an approximately 22% reduction in CL/F, while a doubling of AST from 60 U/L corresponded to an approximately 7% reduction. These findings are biologically plausible, as tacrolimus is primarily metabolized by hepatic CYP3A enzymes, and impaired graft function during the early postoperative period would be expected to reduce metabolic capacity (Moes et al., 2016).

Among the transaminases, AST rather than ALT was retained as the covariate on CL/F. Because ALT and AST are highly correlated markers of hepatocellular injury, their concurrent inclusion would have introduced statistical redundancy. This choice is consistent with prior population PK studies of tacrolimus conducted following liver transplantation, in which AST, rather than ALT, was identified as a significant covariate on apparent clearance (Cai et al., 2022).

Age was included as a covariate on V/F, with the exponent fixed at 0.16 as the covariance step failed to converge when the parameter was estimated freely. Although the covariate effect was modest (ΔOFV = −2.21), age was retained based on biological plausibility; the fraction of total body water declines progressively over the first years of life, and this developmental change would be expected to influence the apparent volume of distribution independently. In fact, body weight was initially evaluated as a significant covariate for pediatric modeling (Anderson and Holford, 2008) but produced a biologically implausible negative exponent on V/F (Supplementary Tables S1, S2), likely reflecting confounding due to perioperative fluid shifts and edema.

Age-dependent maturation of hepatic enzymes has been proposed as a determinant of clearance in pediatric populations, most recently in a semi-mechanistic model of busulfan disposition in children undergoing hematopoietic cell transplantation (Cao et al., 2025). Even though age could be a promising covariate for clearance, the metabolizing organ after liver transplantation is the graft rather than the recipient’s own liver, so neither recipient age nor recipient CYP3A5 genotype describes the metabolic phenotype of the transplanted organ. In hematopoietic cell transplantation, by contrast, the recipient liver is retained, and post-menstrual age remains a meaningful descriptor of enzyme maturation. On the other hand, our analysis was confined to the first 11 postoperative days, during which clearance was dominated by the acute recovery of graft function (Cai et al., 2022). This process would be more affected by TBIL and AST than by recipient characteristics. Consistent with this, the inter-occasion variance in clearance was largest at POD 2 (CV 123.8%) and declined thereafter. Age was tested on CL/F but did not improve the fit (ΔOFV = −0.281 for CL/F) and was therefore retained only on V/F.

The estimated variance structure of IOV on CL/F is the most clinically informative feature of the model. Inter-occasion variability in clearance was greatest at the first trough measurement after tacrolimus initiation (CV 123.8%), fell by more than an order of magnitude to a minimum on postoperative days 5–6 (CV 10.7%), and rose again by postoperative days 9–11 (CV 38.8%). This trajectory suggests three phases of tacrolimus disposition during the early postoperative period: an acutely unstable phase at initiation, a stabilization phase as graft function recovers, and a phase of renewed fluctuation in the second postoperative week. The initial phase reflects intensive perioperative management, including fluid resuscitation, transfusions, hemodynamic instability, and rapidly changing graft function (Efune et al., 2023). The stabilization phase likely corresponds to resolution of acute postoperative changes and improving graft function, while the later fluctuation phase may reflect immunosuppressive adjustments, steroid tapering, and evolving postoperative complications. The first two phases parallel the resolution of graft dysfunction captured by the covariate model, whereas the late increase is not explained by total bilirubin or AST, both of which continued to fall over the same interval; its determinants remain to be established.

However, the substantial η-shrinkage observed for the inter-individual variability (0.97 for CL/F and 0.81 for V/F) limits the interpretability of individual empirical Bayes estimates. Although it does not by itself compromise the predictive performance according to the estimated results of goodness-of-fit diagnostic plots, occasion-stratified pcVPC and individual prediction by Monte Carlo simulations, high η-shrinkage may be a consequence of the sampling design rather than a deficiency of the structural model. In a routine therapeutic drug monitoring setting, each postoperative occasion contributed only one or two trough measurements per subject, which does not provide sufficient information to separate a subject’s time-invariant clearance deviation from occasion-specific fluctuations. Furthermore, patients in the early postoperative period frequently undergo interventions that acutely alter drug disposition, including volume resuscitation, transfusion of blood products or albumin, and modification of concomitant medications. Such interventions can introduce short-term variability in the physiological determinants of tacrolimus disposition.

Consequently, most of the individual information was absorbed into the inter-occasion variability terms, which showed considerably lower shrinkage (0.31–0.85). The high shrinkage for V/F additionally reflects the trough-enriched sampling, which carries limited information of the distribution phase. Therefore, it should be emphasized that individual empirical Bayes estimates of clearance and volume of distribution should not be interpreted in isolation, but rather with the corresponding occasion-specific random effects. In fact, this finding is consistent with the central premise of this analysis: in the immediate postoperative period, clearance is governed predominantly by the evolving state of the graft rather than by stable individual traits, and dose individualization is therefore most appropriately driven by occasion-specific data.

The effect of the graft function (TBIL and AST) on dose requirement is quantified in Table 7. The dose required to maintain a trough of 10 ng/mL rose 2.1-fold between POD 2 and POD 9–11, indicating that a dose appropriate at initiation becomes insufficient within days as graft function recovers. The equation describes a maintenance dose, and its steady-state assumption is not met at the earliest occasions. Tacrolimus was initiated on POD 1, so only two doses had been administered by the first sampling occasion, and with a typical half-life exceeding 24 h the drug was still accumulating. The observed median trough of 4.0 ng/mL at POD 2 is closely reproduced by the model when accumulation is accounted for (predicted as 4.2 ng/mL), and the proportion of the steady-state trough attained rose from 46% at POD 2%–88% by POD 5–6 (Supplementary Table S3), confirming that early subtherapeutic exposure reflects incomplete accumulation rather than model misspecification.

Based on these random effects, predictability of Bayesian forecasting based on the constructed PK model was examined. According to Table 8, individual clearance estimates obtained by MAP estimation from a single POD 2 trough deviated from the covariate-based typical value by −62% to +134% in the four representative subjects, and the discrepancy was not predictable from the covariate values: the subject with the largest upward correction had preserved hepatic function (TBIL 1.9 mg/dL, AST 94 U/L), whereas the subject with the largest downward correction had extreme transaminase elevation (AST 6656 U/L) yet a trough nearly three-fold above the covariate-based prediction.

Quantitatively, according to Table 9, a single trough reduced the mean absolute error of the next-occasion prediction by approximately one-fifth and two troughs by roughly one-third, with coverage of the 90% prediction interval remaining close to nominal throughout (87.0%–92.6%), indicating that the improvement was not obtained at the expense of calibration. Predictive performance was evaluated for the third and fourth postoperative days, the occasions at which consecutive measurements were available in a sufficient number of subjects. The incremental value of additional sampling at later occasions, when inter-occasion variability in clearance is an order of magnitude smaller, is expected to be correspondingly smaller; the evaluation therefore addresses the period in which individualization is most difficult and most consequential.

A single early measurement informs the time-invariant component of clearance but carries no information about the deviation of the occasion being predicted; with an inter-occasion CV of 123.8% at POD 2, most of what is observed on that occasion is not transferable. The benefit therefore accrues as measurements accumulate across occasions, which is precisely the pattern of routine therapeutic drug monitoring in this setting. This also dictates how the inter-occasion deviation should be handled when projecting forward: retaining an estimated κ for a dose to be given at a subsequent occasion would overstate the confidence of the prediction, so κ was reset to its expected value of zero on each occasion boundary, with the associated uncertainty carried by the inter-occasion variance of the upcoming occasion.

This structure has a direct consequence for dose individualization. When a concentration is measured, the deviation it reveals must be allocated between the time-invariant individual effect and the deviation specific to that occasion, and the allocation is affected by their relative variances. At the first occasion, where inter-occasion variability was largest (CV 123.8%), most of the observed deviation is attributed to the occasion itself rather than to the individual, and since the occasion-specific term is reset when projecting forward, comparatively little of that information is carried into the next occasion. Early measurements are therefore less informative about subsequent occasions than their timing might suggest, and the value of therapeutic drug monitoring accrues as measurements accumulate across occasions rather than from any single early sample.

The population PK model was further developed to demonstrate the MIPD of tacrolimus and to realize a clinically available platform. Initial dosing may continue to follow conventional body weight–based strategies, followed by Bayesian updating as additional TDM data become available. This approach enables dynamic dose adaptation across postoperative days and may improve exposure targeting compared with conventional trough-based dosing strategies under sparse sampling conditions.

Other limitations of this modeling include the single-center retrospective design, which may limit generalizability. External validation in independent cohorts is required. In addition, sparse trough-based sampling limited estimation of absorption parameters, which were fixed to literature values, and may have contributed to increased η-shrinkage. Finally, key covariates such as CYP3A5 genotype and co-medications were not available, which may account for residual unexplained variability.

In conclusion, this study developed a population PK model of tacrolimus in pediatric liver transplant recipients younger than 36 months during the immediate postoperative period. Apparent clearance was described by total bilirubin and AST as dynamic markers of graft function, and inter-occasion variability was estimated separately for each of six postoperative occasions, revealing a more than tenfold change in within-subject variability over 11 days. The model used Bayesian forecasting to improve prediction of the next occasion as trough measurements accumulated. These results imply that tacrolimus dose individualization during the immediate postoperative period is most appropriately driven by occasion-specific data and provide a quantitative basis for model-informed precision dosing. Prospective validation in independent cohorts and integration into clinical decision-support systems remain necessary before routine implementation.

## Data Availability

The original contributions presented in the study are included in the article/Supplementary Material, further inquiries can be directed to the corresponding author.
