# Supplementary material for: Population pharmacokinetic modeling of tacrolimus using real-world data for precision dosing in pediatric liver transplant recipients younger than 36 months during the immediate postoperative period
Source: Front Pharmacol. 2026 Sep 23;17:1896778. doi: 10.3389/fphar.2026.1896778 (PMC13642222; doi:10.3389/fphar.2026.1896778)
Supplement: Supplementary file 1 [file Supplementaryfile1.docx]

**Supplementary Figure 1.** **Individual concentration-time profiles of tacrolimus in 92 pediatric liver transplant recipients**

Each panel represents concentration-time profile of each subject included in the PK analysis. Open circles denote observed concentrations (DV), blue dashed lines denote population predicted values (PRED), and red solid lines denote individual predicted values (IPRE) from the final population pharmacokinetic model. The $x$-axis shows time after the first dose (h) and the $y$-axis shows tacrolimus whole-blood concentration (ng/mL).
