# Supplementary material for: Population pharmacokinetic modeling of tacrolimus using real-world data for precision dosing in pediatric liver transplant recipients younger than 36 months during the immediate postoperative period
Source: Front Pharmacol. 2026 Sep 23;17:1896778. doi: 10.3389/fphar.2026.1896778 (PMC13642222; doi:10.3389/fphar.2026.1896778)
Supplement: Supplementary file 2 [file Supplementaryfile2.docx]

**Supplementary Figure 2.** **Individual concentration–time profiles generated by Monte Carlo simulation using the final population PK model**

For each subject, 250 concentration-time profiles were simulated using the observed dosing history and time-varying covariates, with all final model parameters fixed to their estimated values. The shaded band represents the 5th–95th prediction interval of the simulated concentrations at each time point, and the solid line represents the simulated median. Open blue circles indicate observed tacrolimus concentrations. Subject identifiers were anonymized. Profiles for all 92 subjects included in the analysis are provided in Supplementary Figure 1.
