# Supplementary material for: Population pharmacokinetic modeling of tacrolimus using real-world data for precision dosing in pediatric liver transplant recipients younger than 36 months during the immediate postoperative period
Source: Front Pharmacol. 2026 Sep 23;17:1896778. doi: 10.3389/fphar.2026.1896778 (PMC13642222; doi:10.3389/fphar.2026.1896778)
Supplement: Supplementary file 3 [file Table1.docx]

**Supplementary Table 1.** **Initial screening of covariate analysis for typical apparent clearance and volume of distribution**

| **Covariate** | **PE** | **SE** | **OFV** | **ΔOFV** | **AIC** | **ΔAIC** |
| --- | --- | --- | --- | --- | --- | --- |
| ***Initial covariate screening for TV(CL/F)*** | | | | | | |
| Base | | | 2695.315 | — | 2721.822 | — |
| **Total bilirubin** | **−0.397** | **0.046** | **2608.613** | **-86.702** | **2637.199** | **-84.623** |
| **ALT** | **−0.290** | **-** | **2632.432** | **-62.883** | **2661.018** | **-60.804** |
| **AST** | **−0.215** | **-** | **2659.378** | **-35.937** | **2687.964** | **-33.858** |
| **Serum albumin** | **0.507** | **0.157** | **2682.003** | **-13.312** | **2710.589** | **-11.233** |
| **Serum creatinine** | **−0.296** | **0.121** | **2684.591** | **-10.724** | **2713.176** | **-8.646** |
| Graft weight | 0.249 | 0.188 | 2693.657 | -1.658 | 2722.243 | 0.421 |
| Body weight | −0.221 | 0.218 | 2694.014 | -1.301 | 2722.599 | 0.777 |
| Hematocrit | −0.108 | 0.195 | 2694.898 | -0.417 | 2723.484 | 1.662 |
| Age | 0.060 | 0.121 | 2695.034 | -0.281 | 2723.619 | 1.797 |
| Hemoglobin | 0.047 | 0.142 | 2695.148 | -0.167 | 2723.733 | 1.911 |
| Sex (categorical) | 0.022 | 0.109 | 2695.274 | -0.041 | 2723.859 | 2.037 |
| Age at operation | 0.021 | 0.122 | 2695.280 | -0.035 | 2723.866 | 2.044 |
| ABO type (categorical) | −0.001 | 0.040 | 2695.314 | -0.001 | 2723.900 | 2.078 |
| ***Initial covariate screening for TV(V/F)*** | | | | | | |
| Base | | | 2695.315 | — | 2721.822 | — |
| Body weight | −0.151 | 0.085 | 2692.759 | -2.556 | 2721.345 | -0.477 |
| ALT | −0.069 | 0.079 | 2693.567 | -1.748 | 2722.152 | 0.330 |
| Total bilirubin | −0.110 | 0.175 | 2694.030 | -1.285 | 2722.616 | 0.794 |
| Age at operation | 0.108 | 0.127 | 2694.137 | -1.178 | 2722.723 | 0.901 |
| Age | 0.105 | 0.129 | 2694.214 | -1.101 | 2722.800 | 0.978 |
| Graft weight | −0.137 | 0.175 | 2694.734 | -0.581 | 2723.320 | 1.498 |
| AST | −0.027 | 0.110 | 2695.071 | -0.244 | 2723.656 | 1.834 |
| Serum creatinine | 0.010 | 0.111 | 2695.302 | -0.013 | 2723.888 | 2.066 |
| Serum albumin | 0.024 | 0.069 | 2695.264 | -0.051 | 2723.850 | 2.028 |
| Sex (categorical) | 0.022 | 0.109 | 2695.274 | -0.041 | 2723.859 | 2.037 |
| Hemoglobin | 0.017 | 0.053 | 2695.271 | -0.044 | 2723.857 | 2.035 |
| ABO type (categorical) | −0.001 | 0.040 | 2695.314 | -0.001 | 2723.900 | 2.078 |

TV(CL/F), typical apparent clearance; TV(V/F), typical volume of distribution; PE, point estimate; SE, standard error; OFV, objective function value; AIC, corrected Akaike information criterion.

Base model objective function value (OFV) was 2695.315 and corrected Akaike information criterion (AIC) was 2721.822. ΔOFV and ΔAIC were calculated as the difference between each candidate single-covariate model and the base model. Covariates producing a reduction in OFV exceeding 3.84 (corresponding to p < 0.05 with 1 degree of freedom) are highlighted in green and were considered statistically significant in the univariate screening. Continuous covariates were tested using a power model centered at clinically meaningful reference values; categorical covariates (gender, ABO type) were tested as a multiplicative fractional shift on the typical parameter value. Point estimates (PE) and standard errors (SE) are presented after initial covariate screening and rounded to four decimal places. Standard errors are omitted when estimation is failed because of numerical problems.
