# Supplementary material for: Population pharmacokinetic modeling of tacrolimus using real-world data for precision dosing in pediatric liver transplant recipients younger than 36 months during the immediate postoperative period
Source: Front Pharmacol. 2026 Sep 23;17:1896778. doi: 10.3389/fphar.2026.1896778 (PMC13642222; doi:10.3389/fphar.2026.1896778)
Supplement: Supplementary file 4 [file Table2.docx]

**Supplementary Table 2.** **Subsequent covariate screening for TV(CL/F) based on the TV(CL/F) ~ TBIL model, and for TV(V/F) based on the TV(CL/F) ~ TBIL + AST model**

| **Covariate added** | **Model** | **PE** | **SE** | **OFV** | **ΔOFV** | **AIC** | **ΔAIC** |
| --- | --- | --- | --- | --- | --- | --- | --- |
| ***Second covariate screening of TV(CL/F)*** | | | | | | | |
| **—** | **TV(CL/F) ~ TBIL** | **−0.397** | **0.046** | **2608.613** | **—** | **2637.199** | **—** |
| ALB | TV(CL/F)  ~ TBIL + ALB | 0.453 | 0.158 | 2597.295 | -11.318 | 2627.965 | -9.234 |
| ALT | TV(CL/F)  ~ TBIL + ALT | −0.141 | - | 2598.481 | -10.132 | 2629.152 | -8.047 |
| **AST** | **TV(CL/F)**  **~ TBIL + AST** | **−0.108** | **0.049** | **2600.557** | **-8.056** | **2631.227** | **-5.972** |
| SCR | TV(CL/F)  ~ TBIL + SCR | −0.197 | 0.105 | 2603.373 | -5.240 | 2634.043 | -3.156 |
| ***Covariate screening for TV(V/F) based on TV(CL/F) ~ TBIL + AST*** | | | | | | | |
| AGE | TV(V/F) ~ AGE | 0.156 | 0.103 | 2598.348 | -2.209 | 2631.109 | -0.118 |
| WT | TV(V/F) ~ WT | −0.149 | 0.109 | 2598.654 | -1.903 | 2631.415 | 0.188 |

TV(CL/F), typical apparent clearance; TV(V/F), typical apparent volume of distribution; ALB, serum albumin; ALT, alanine aminotransferase; AST, aspartate aminotransferase; SCR, serum creatinine; TBIL, total bilirubin; WT, body weight; PE, point estimate; SE, standard error; AIC, corrected Akaike information criterion; OFV, objective function value.

TBIL was selected as the reference model after initial covariate analysis of TV(CL/F). Other covariates (ALB, ALT, AST and SCR) were subsequently included to finalize the covariate model of TV(CL/F). ΔOFV and ΔAIC were calculated as the difference between each two-covariate model and the TBIL-only model. TBIL + AST (highlighted in green) was selected as the final model based on the complementary biological role of the two markers in capturing acute graft function during the early postoperative period. TBIL reflects integrative graft excretory and synthetic function, while AST serves as a sensitive marker of acute hepatocellular injury. Although models containing ALB or ALT yielded modestly lower OFV and AIC values, these covariates were considered less directly representative of acute graft injury relevant to tacrolimus pharmacokinetic variability in this clinical phase, and serum albumin measurements can additionally be confounded by exogenous albumin administration commonly used in the early postoperative period. SCR was not included in the final model due to the statistical and pharmacological considerations. For statistical reasons, TV(CL/F) ~ TBIL + SCR resulted in higher OFV and AIC values than TV(CL/F) ~ TBIL + AST model. For pharmacological reasons, tacrolimus is predominantly metabolized hepatically rather than by renal excretion, and therefore renal function is not a direct determinant of its clearance. Furthermore, taaffectsus itself can elevate serum creatinine through calcineurin inhibitor nephrotoxicity, which in turn reversely affect causation. The final model included TBIL and AST as covariates affecting TV(CL/F), highlighted in green. Point estimates (PE) and standard errors (SE) are presented after initial covariate screening and rounded to four decimal places. Standard errors are omitted when estimation is failed because of numerical problems. For TV(CL/F) ~ TBIL, the reported PE and SE represent the effect of TBIL. For the remaining covariates (ALB, ALT, AST, and SCR), PE and SE represent the effects of each covariate when added individually to the TBIL-based model. Pediatric-specific covariate screening for TV(V/F) was done based on TV(CL/F) ~ TBIL + AST model.
