# Supplementary material for: Population pharmacokinetic modeling of tacrolimus using real-world data for precision dosing in pediatric liver transplant recipients younger than 36 months during the immediate postoperative period
Source: Front Pharmacol. 2026 Sep 23;17:1896778. doi: 10.3389/fphar.2026.1896778 (PMC13642222; doi:10.3389/fphar.2026.1896778)
Supplement: Supplementary file 5 [file Table3.docx]

**Supplementary Table 3.** **Attainment of steady state across pharmacokinetic occasions in the early postoperative period**

| **OCCPK** | **POD** | **No. of samples** | **Model-predicted trough accounting for accumulation (ng/mL)** | **Observed trough (ng/mL)** | **Proportion of steady-state trough attained (%)** |
| --- | --- | --- | --- | --- | --- |
| 1 | 2 | 77 | 4.2 | 4.0 | 46 |
| 2 | 3 | 74 | 6.8 | 7.5 | 63 |
| 3 | 4 | 79 | 8.3 | 9.2 | 85 |
| 4 | 5–6 | 141 | 9.2 | 9.6 | 88 |
| 5 | 7–8 | 147 | 9.1 | 8.4 | 99 |
| 6 | 9–11 | 213 | 8.6 | 8.4 | 101 |

OCCPK, pharmacokinetic occasion; POD, postoperative day.

For each subject, the trough concentration expected at the sampling time of the occasion was computed by superposition over the actual dosing history using the typical apparent clearance and volume of distribution implied by that subject's covariates and expressed as a proportion of the steady-state trough for the same dose and parameters. Values are shown as occasion medians. The close agreement between the accumulation-based prediction and the observed trough, together with the progressive rise toward steady state, indicates that the subtherapeutic concentrations observed at the earliest occasions reflect incomplete drug accumulation rather than model misspecification: tacrolimus was initiated on postoperative day 1, and with a typical half-life exceeding 24 hours the drug was still accumulating at the first sampling occasions.

In the early phase of drug administration, steady state is not achieved, and the rule of superposition is applied to predict the trough concentration, as shown in Equation ST3-1.

$$\begin{aligned} C\left( t \right)=\sum_{i (t_{i}\leq T)} \frac{D_{i}\cdot k_{a}}{V\cdot(k_{a}-k)}\cdot\left( e^{-k(T-t_{i})}-e^{-k_{a}(T-t_{i})} \right)\#\left( ST3-1 \right) \end{aligned}$$

where $D_{i}$: the $i$-th dose, $t_{i}$: the time of $i$-th dose, $k$: the elimination rate constant, $k_{a}$: the absorption rate constant fixed at 4.5 $h^{-1}$, $T$: the time at observation.

If the drug is administered repeatedly, it will reach a steady state. Equation ST3-2 denotes the trough concentration when the steady state is reached after sufficient administration of the drug.

$$\begin{aligned} C_{ss,trough}=\frac{D_{i}\cdot k_{a}}{V\cdot(k_{a}-k)}\cdot\left( \frac{e^{-k\tau}}{1-e^{-k\tau}}-\frac{e^{-k_{a}\tau}}{1-e^{-k_{a}\tau}} \right)\#\left( ST3-2 \right) \end{aligned}$$

where $\tau$: the dosing interval (h). Note that $\frac{e^{-k\tau}}{1-e^{-k\tau}}$ and $\frac{e^{-k_{a}\tau}}{1-e^{-k_{a}\tau}}$ are $\sum_{n=0}^{\infty} e^{-kn\tau}$ and $\sum_{n=0}^{\infty} e^{-k_{a}n\tau}$, respectively.

The fraction of the steady-state trough concentration attained at the time of sampling is calculated as in Equation ST3-3.

$$\begin{aligned} \frac{C(t)}{C_{ss, trough}}\times100 \left( \% \right)\#\left( ST3-3 \right) \end{aligned}$$
